# Outcomes of a Multidisciplinary Clinic in Evaluating Recurrent *Clostridioides difficile* Infection Patients for Fecal Microbiota Transplant: A Retrospective Cohort Analysis

**DOI:** 10.3390/jcm8071036

**Published:** 2019-07-16

**Authors:** Jae Hyun Shin, Ashley S. Chaplin, R. Ann Hays, Glynis L. Kolling, Sheila Vance, Richard L. Guerrant, Laurie Archbald-Pannone, Cirle A. Warren

**Affiliations:** 1 Department of Medicine, Division of Infectious Diseases and International Health, University of Virginia, Charlottesville, VA 22903, USA; 2 Novant Health, Winston-Salem, NC 27103, USA; 3 Department of Medicine, Division of Gastroenterology and Hepatology, University of Virginia, Charlottesville, VA 22903, USA; 4 Department of Gastroenterology, Veterans Affairs Connecticut Healthcare System, West Haven, CT 06516, USA; 5 Department of Medicine, Division of General, Geriatric, Palliative and Hospital Medicine, University of Virginia, Charlottesville, VA 22093, USA

**Keywords:** *Clostridium difficile* infection, *Clostridioides difficile* infection, fecal microbiota transplant, recurrent *C. difficile*

## Abstract

Fecal microbiota transplantation (FMT) has been shown to be an effective treatment for recurrent *Clostridioides difficile* infections (rCDIs). We assessed the benefits of a multidisciplinary *C. difficile* clinic for screening FMT eligibility in patients with rCDI. Patients seen at the University of Virginia Complicated *C. difficile* Clinic (CCDC) underwent comprehensive evaluation for possible FMT. Patients were eligible for FMT if there was history of greater than two episodes of rCDI. Patients were evaluated for the outcome after evaluation in the clinic. A total of 113 patients were evaluated: 77 were eligible for FMT, of which 25 patients did not undergo FMT. The rate of recurrence at three months and all-cause mortality were 4.5% and 7% for patients who received FMT and 16.7% and 12.5% for eligible patients who did not receive FMT. There were 36 patients who were not eligible for FMT, with two or fewer recurrences and a recurrence rate of 8.8% and all-cause mortality of 6%. One in three patients screened for FMT had a nutritional deficiency diagnosed, with zinc deficiency being most common (20%). Additional diagnoses, including inflammatory bowel disease, were made during the evaluation. FMT is a highly effective treatment for rCDI, most notably in patients with multiple recurrences. A systematic approach for evaluating patients with rCDI helps identify patients who benefit most from FMT and those who have other conditions.

## 1. Introduction

*Clostridioides* (*Clostridium*) *difficile* (Hall and O’Toole 1935) Lawson et al. 2016 infection (CDI) is the top urgent threat for drug-resistance in the United States [1]. There are 29,000 deaths from CDI each year in the United States alone [1,2]. CDI recurs in 20% of patients and cycles of recurrent infection are debilitating with up to 65% of those who recur once experiencing multiple recurrences [3]. Despite increased infection control and prevention initiatives, CDI is still the most common nosocomial infection, representing 12% of health-care associated infections and causing $4.8 billion in excess health-care costs [4,5].

Fecal microbiota transplantation (FMT) has been shown to be the most effective treatment for recurrent CDI (rCDI) [6,7,8,9,10]. Current guidelines from the Infectious Diseases Society of America now recommend FMT for second and subsequent recurrences of CDI (after appropriate antibiotic treatments for two or more recurrences) and subsequent rCDI [11]. Likewise, 2013 guidelines from the American College of Gastroenterology recommend FMT for patients who have experienced three or more recurrences after a pulsed vancomycin course [12].

We established an interdisciplinary Complicated *C. difficile* Clinic (CCDC) to provide a comprehensive medical evaluation of patients with rCDI and to offer FMT for appropriate patients. In this study, we present the clinical outcomes from our initial cohort of patients evaluated in this clinic (2012–2015) that were treated with FMT or standard therapy.

## 2. Methods

### 2.1. Study Setting

The CCDC is a referral clinic attended by a multidisciplinary team consisting of a gastroenterologist, geriatrician, infectious diseases physician, and/or physician’s assistant. The interdisciplinary team held weekly conferences to review criteria and discuss patients who were immunocompromised or medically complex. Patients underwent extensive history and laboratory evaluation for rCDI and other potential causes of chronic or persistent diarrhea at the initial clinic visit per protocol. The laboratory studies ordered at the initial visit are listed in Table 1. We performed the tests regardless of the symptomatology or medical history, to obtain baseline conditions prior to potential FMT. Because of the still many uncertainties on the intestinal or extraintestinal and both short- and long-term effects of FMT, we opted to be as comprehensive as possible in these laboratory procedures. Of particular concern were Epstein-Barr Virus (EBV) or Cytomegalovirus (CMV) seronegative patients who may be either immunocompetent or have undiagnosed immunocompromising conditions that may acquire the disease post-FMT from a seropositive donor, a potential issue that has never been addressed directly in previous literature. The plan from our clinic was to modify our procedure as appropriate based on what we learned from this initial cohort of patients. Patients also received CDI-specific medical and lifestyle education regarding antibiotic use, proton pump inhibitors, and personal and environmental hygiene (Table 2). As per current Food and Drug Administration regulations, FMT was explained to be an investigational treatment and potential risks were discussed prior to gaining informed consent for FMT from eligible patients [13].

### 2.2. Study Design

We performed a retrospective cohort analysis of patients evaluated in the CCDC from June 2012 to March 2015. The research protocol was approved by the Institutional Review Board at the University of Virginia. We reviewed the electronic medical record of each patient to collect patient demographics, CDI history, laboratory tests (blood and stool), and clinical data from encounter notes.

### 2.3. Study Population

All patients seen at the CCDC were included. Eligibility for FMT was determined using the guidelines published by Surawicz et al. [12] Patients were considered eligible for FMT if they had experienced three or more CDI recurrences despite appropriate treatment for previous episodes of CDI, including at least one treatment being a prolonged vancomycin tapering course [12]. Patients could be asymptomatic or with active recurrent disease. Immunocompromised patients who fulfilled the following criteria were excluded: patients with neutropenia (absolute neutrophil count (ANC) < 500), patients who had undergone solid organ or bone marrow transplantation within the prior year unless the patient’s primary oncologic or transplant team was in agreement that the benefit outweighs the risk of the procedure, or patients whose the primary oncologic or transplant team were not in agreement with FMT. Patients were also excluded if they were unable to discontinue non-CDI antibiotics prior to, during, and at least one week after FMT. Prior use of alternative strategies for recurrent CDI such as bezlotoxumab, probiotics, investigational agents, and failed FMT did not exclude patients from FMT. Likewise, patients with underlying gastrointestinal disorders such as IBD, IBS, and motility disorders were not excluded as long as the CDI episodes were appropriately documented and the risk of persistent symptoms or partial response to treatment were discussed.

### 2.4. FMT Procedure

We established a laboratory supervised by an experienced faculty microbiologist to manage stool procurement, storage, and processing. The directed donor specimens were processed in this laboratory and universal donor specimens were prescreened and purchased from OpenBiome (Boston, MA USA). These stool samples were labeled, stored frozen, and processed in a dedicated Biosafety Level-2 laboratory in a biological safety cabinet and hand delivered to the GI Endoscopy suite at the time of FMT. Symptomatic patients were treated with a tapering course of vancomycin, which was stopped 2 days prior to the scheduled FMT. FMT was primarily delivered by colonoscopy (CSY). FMT was offered by upper gastrointestinal endoscopy in the distal duodenum (EGD) or percutaneous gastrostomy tube (PEG) after pretreatment with metoclopramide in special circumstances. Random biopsies and/or targeted intestinal biopsies were obtained at the discretion of the gastroenterologist.

### 2.5. Patient Follow-Up

After FMT, patients were followed closely with telephone contact at 1 week (range 2–10 days), 1 month (30 days ± 14), 3 months (90 days ± 30), 6 months (180 days ± 30), and 12 months (360 days ± 30) after the procedure. If not eligible or appropriate for FMT, patients were observed for recurrence if asymptomatic and treated with standard antibiotic therapy if with active disease. All patients were instructed to contact the clinic staff with recurrent diarrhea or concern for rCDI. For the non-FMT cohort, median duration from a clinic visit to survey follow-up was approximately 348 days (range 96–932 days). These patients were surveyed via telephone regarding overall health and quality of life using the same scale for the FMT cohort. If we failed to contact these patients after three attempts, they were deemed lost to the follow-up.

### 2.6. Study Outcomes

Primary outcomes of clinical interest were CDI recurrence within three months of FMT for patients who received FMT or CDI recurrence within three months of the initial visit for patients not treated with FMT. CDI recurrence was defined as return of diarrhea with a stool sample positive for *C. difficile* toxin. Diarrhea was defined as having loose stool taking the shape of the receptacle and occurring more than three times per day. Testing for *C. difficile* toxin at the University of Virginia Health System were performed by toxin B gene PCR (Xpert Cepheid, CA, USA). Testing outside the University of Virginia System was not uniform, but followed the clinical diagnostic decision by the treating physician. The secondary outcomes were long-term success of FMT for treatment of rCDI (12 months), changes in overall health (energy level, weight), gastrointestinal symptoms (abdominal pain, appetite, bowel movements), and occurrence of acute illness, new medications, hospitalization, and surgery. Overall health and symptoms were assessed subjectively as “worse/decreased”, “unchanged” or “improved/increased”.

### 2.7. Statistical Analysis

Chi-square analysis, Fisher’s exact test, or the Student’s t test was used for analysis. A 2-tailed P value of 0.05 was considered statistically significant (SPSS version 23.0, Armonk, NY, USA: IBM Corp.). For recurrences, recurrence-free survival was used, which was defined as the time from FMT to removal from the study due to a recurrence for the FMT group, and the time from the initial visit to removal from the study due to a recurrence for the non-FMT group. Kaplan–Meier plots were generated and analyzed by Mantel–Cox log-rank test using Graphpad Prism 7 software (La Jolla, CA, USA).

## 3. Results

### 3.1. Cohort Demographics

We evaluated 113 patients (mean age 64 years, range 20–92 years) in the clinic during the study period. Cohort demographics are shown in Table 3. Patients treated with FMT and those not treated with FMT were demographically similar, with the exception of pulmonary disorders (more common in the latter). Among the 77 patients who met the criteria for FMT of three or more CDI recurrences, 52 (67.5%) were treated with FMT (*n* = 50 via CSY, *n* = 1 via EGD, *n* = 1 via PEG). Among the 25 (32.5%) patients that met the criteria with three or more CDI recurrences, procedure was deferred due to patient preference and/or safety concerns from co-morbidities in the context of FMT (Table 4). There were 36 patients who did not meet criteria for FMT due to having had less than three recurrences.

### 3.2. Primary Outcomes

**Figure 1:**
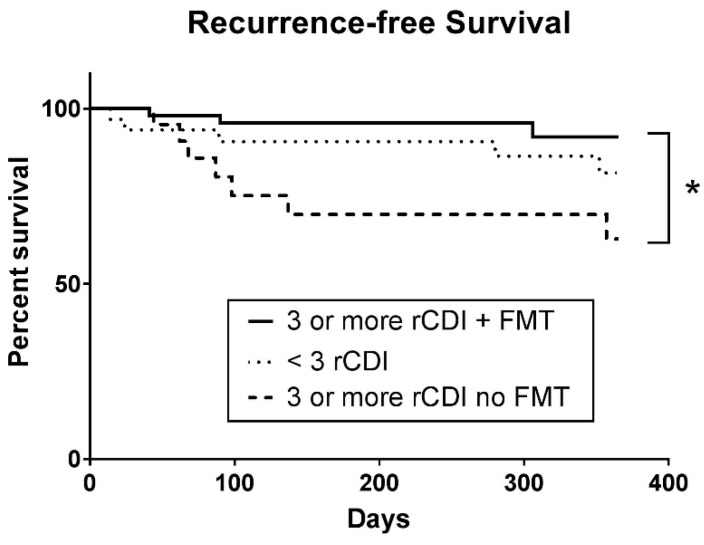
Recurrence-free survival in FMT and non-FMT groups. Recurrence-free survival analysis was performed using the Mantel–Cox log-rank test. The following three groups were compared: patients with three or more recurrent CDIs (rCDIs) who underwent FMT, patients with less than three rCDIs and therefore ineligible for FMTs, and patients with three or more rCDIs who deferred FMT due to other reasons.

Forty-four of the 52 patients who received FMT had information available at the three-month follow-up. Out of the 44 patients, two patients experienced CDI recurrences, on day 41 and day 90, respectively, resulting in a 4.5% recurrence rate (Figure 1). At 12 months, 24 patients had information available for follow-up. One additional recurrence occurred on day 306, bringing the total number of recurrences at 12 months in the FMT group to 12.5%. Three patients died within the study period after receiving FMT resulting in an all-cause mortality of 7% (death at 1 month, *n* = 1; 12 months, *n* = 2). The cause of death was not directly related to FMT or recurrent CDI, i.e., cardiac arrest secondary to pulmonary embolism after a hip fracture, congestive heart failure, or ileus. Twenty-four of the 25 patients who met the criteria for FMT of three or more CDI recurrences but deferred the procedure had follow-up information available. Four patients experienced a recurrence within three months (16.7% recurrence rate), on days 44, 62, 68, and 87. After the final survey, seven patients had recurrences in the first 12 months due to three additional recurrences occurring on days 98, 137, and 357, resulting in a 29.2% long-term recurrence rate, as well as one without a clear date of the recurrence. Six of the patients who experienced recurrences eventually underwent FMT, as well as one patient who did not have further documented recurrence. Three patients died, two from causes unrelated to CDI and one unknown, resulting in an all-cause mortality of 12.5%. Thirty-four of the 36 patients who were initially ineligible for FMT due to having fewer than three recurrences had follow-up information available. Five of 34 patients (14.7%) had recurrences, three within 3 months on days 14, 24, and 89 (3 months recurrence rate 8.8%), and two more, on days 280 and 352, within 12 months. Two eventually underwent FMT after a recurrence as they now fulfilled the criteria for FMT. Two died of non-CDI-related causes, resulting in an all-cause mortality of 6%.

Using the Mantel–Cox log-rank test, there was a statistically significant difference in survival analysis between the FMT group and the FMT-eligible but deferred group (*p* = 0.0030). Due to the lack of information on the date of recurrence, one patient with recurrence in the FMT eligible but deferred group was not included in the analysis.

### 3.3. Secondary Outcomes

**Figure 2:**
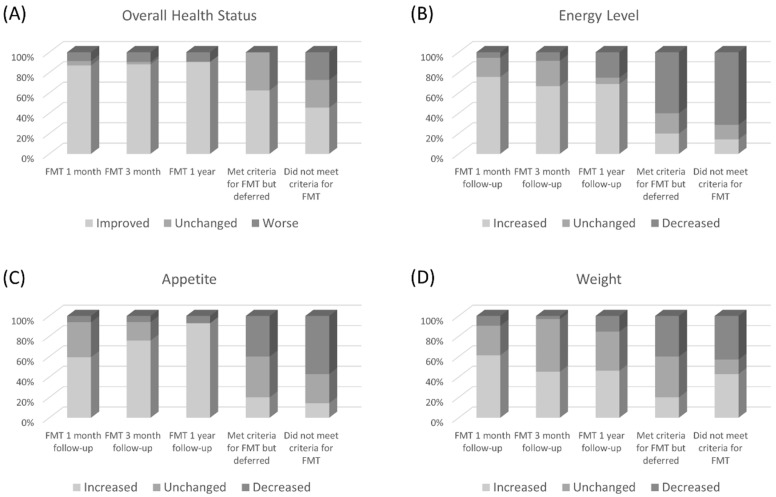
Secondary outcomes in follow-up for FMT and non-FMT groups.

Up to 90% of the FMT recipients reported an improvement in their overall state of health on follow-up at one, three, and 12 months after the procedure (Figure 2A). Fourteen of the 44 patients who had follow-up were hospitalized within 12 months with three hospitalizations related to rCDI. Patients who did not receive FMT reported significantly less “improved” and more “unchanged” overall state of health. Twelve of the 28 non-FMT patients were admitted ≥ 24 h to a hospital or rehabilitation facility.

In addition to a subjective improvement in overall state of health, similar trends were observed with changes in energy level, appetite, and weight, where non-FMT patients reported more “decreased” rates in these parameters as compared with FMT patients (Figure 2B–D). Information about abdominal pain and bowel movements were also obtained but there were no significant differences noted between the treatment groups.

### 3.4. New Diagnoses Discovered from FMT Work-Up

Thirty-nine of 113 (34.5%) patients were newly diagnosed with a vitamin and/or mineral deficiency: vitamin A (three or 2.7%), vitamin B12 (10 or 8.8%), vitamin D (12 or 10.6%), and zinc (22 or 19.5%). Ten had newly detected thyroid disorders and three had concomitant norovirus infection. The detailed gross and microscopic pathology for patients treated with FMT is shown in Table 5. Thirty-five patients (70%) had colon biopsy, of which five revealed a new diagnosis of inflammatory bowel disease and another five with neoplasms, including one colon adenocarcinoma and four tubular or tubulovillous adenomas.

## 4. Discussion

With our complicated *C. difficile* clinic, we aimed to provide state-of-the-art, multidisciplinary care to our patients suffering from CDI, as well as study the effects of FMT in rCDI. An important aspect of this clinic was that input from three different specialties (gastroenterology, geriatrics, and infectious diseases) were combined to guide diagnostic and therapeutic decisions. We were able to gain valuable insight about the role of FMT in treatment of rCDI by comparing outcomes between the treatment groups.

The most important finding from this study is the value of using screening criteria of three or more recurrences to identify patients most likely to benefit from FMT. When treated with standard non-FMT treatment, the patients who had three or more recurrences had higher recurrence rates (16.7% vs. 8.8%) and mortality rates (12.5% vs. 6%) as compared with patients who had fewer than two recurrences. When this patient population was treated with FMT, the outcome was better, similar to patients with fewer recurrences at recruitment, with similar recurrence rates (4.5% vs. 8.8%) and mortality (7% vs. 6%). The impact of FMT on patients with three or more recurrences was more evident with survival analysis, which showed a statistically significant difference between the patients treated with FMT and the patients treated with standard therapy among patients with three or more recurrences. These findings point to the population with three or more recurrences as the population most likely to benefit from FMT. The literature on FMT has focused on patients with rCDI, with a range of recurrences but with the usual median number of recurrences around three [10,14]. With the success of FMT for rCDI, there has been some thoughts of expanding the indication to patients with fewer recurrences or for initial CDI [15]. The findings in this study confirm that the candidates identified by the three or more recurrence criteria clearly benefit from FMT.

In addition to resolution of rCDI, we showed that patients treated with FMT also reported subjective improvement in their overall health, energy level, appetite, and weight. While perception of overall health and increased energy level may be indirect effects of gastrointestinal function from treated infection, these changes may also be related to extraintestinal effects of the gut flora. Numerous animal studies have demonstrated that the gut microbiome is an integral component of the gut-brain axis, a bidirectional communication pathway that regulates the digestive system and other areas of physiology [16]. Developmental studies in germ-free mice models indicate that microbial colonization influences signaling pathways involved in motor activity and anxiety behavior [17,18]. Moreover, murine studies have demonstrated that diet or exercise-induced metabolic changes and weight effects are transmitted via FMT [16,19]. Interestingly, in our cohort, patients who were ineligible for FMT due to having fewer than two recurrences had subjectively poorer overall health, energy level, appetite, and weight as compared with those who received FMT, even though the recurrence rates were low in both groups. This finding may suggest that the altered microbiome state, required for development of initial CDI episode, also causes worse subjective health states even without causing rCDI.

It was remarkable that up to one-third of the patients who were eligible did not receive FMT due to patient preference or risk of sedation. FMT delivered by oral route may increase application of the procedure as the risks involved with endoscopy are avoided. A recent randomized clinical trial showed that 66% of recipients of oral capsule, versus 44% of colonoscopy delivered FMT, rated their experience “not at all unpleasant” (*p* = 0.01) suggesting that the oral route may be more acceptable to patients [20]. Thus far, the data suggest FMT is safe for treatment of rCDI. However, the optimal route for delivery of FMT, as well as the optimal content to be transplanted (whole stool FMT versus defined FMT), are still not known and need further research.

In addition to access to FMT, the multidisciplinary approach in our CDI-focused clinic provided other benefits. Patients were provided targeted education on *C. difficile* (risk factors, prevention strategies, and treatment options), as well as discussion of lifestyle modifications by clinical *C*. *difficile* experts. The intensive evaluation at the initial visit also led to diagnoses of concomitant infections, potential metabolic disorders or nutritional deficiencies as a result of chronic or persistent diarrhea. Especially notable are the 22 of the 113 patients who were diagnosed with zinc deficiency. Although data from in vitro and animal studies suggest excess zinc worsens CDI [21], our observational clinical data showed an association between zinc deficiency and rCDI that could be improved with zinc replacement [22]. Although further studies are necessary to study the role of micronutrients in CDI pathogenesis, this finding raises the possibility that an initial episode of CDI leads to conditions other than recurrence of infection that causes persistent symptoms and can be corrected by nutritional supplementation. Although not appropriate for all FMT eligible patients, colonoscopy likewise led to new diagnoses such as IBD and colonic malignancy in our cohort (Table 5).

The retrospective nature of this analysis is a limitation of the study. The quality of the data was dependent on accurate recording at the time of the patient encounter, patient recall at follow-up visits or telephone survey, and compliance to the recommended follow-up visits. Due to the retrospective nature of the study, ascertainment of the diagnosis of recurrent CDI post-FMT for patients who were seen outside of our institution was also a limitation. Patients diagnosed in the University of Virginia Health System were diagnosed using PCR, but outside institutions had varying methodologies for diagnosis. These subjects were evaluated on an individual basis, taking into consideration documentation of symptomatology, diagnostic algorithm, response to anti-CDI treatment, and assessment of the clinician who saw the patient.

## 5. Conclusions

While the success of FMT as a treatment for rCDI is now well documented, our experience demonstrates the effectiveness of a multidisciplinary clinic for the evaluation and management of patients with rCDI and a systematic approach for identifying patients who will benefit most from FMT.

## Figures and Tables

**Table 1:** Laboratory studies ordered at initial visit.

Serum
Complete blood count
Comprehensive metabolic panel
Thyroid stimulating hormone
Vitamin A, B12, D and zinc
Immunoglobulin levels
Erythrocyte sedimentation rate and c-reactive protein
Serologies for human immunodeficiency virus, cytomegalovirus, Epstein-Barr virus, human T-cell lymphotropic virus, syphilis, and Strongyloides
Tissue transglutaminase IgA
**Stool**
*Campylobacter*
*Cryptosporidium*
*Giardia*
Norovirus
Shiga toxin
*Clostridioides difficile* toxin
Culture
Ova and parasite
Lactoferrin

**Table 2:** Education provided in clinic for recurrent *Clostridioides difficile* infection.

Objectives
Clean the home with 10% bleach solution.
Exchange toothbrush if kept within six feet of the toilet.
Stop or decrease proton pump inhibitor if possible.
Wash hands with soap and water.
Use separate bathroom if there is more than one bathroom in the house.
Avoid any unnecessary antibiotics.

**Table 3:** Demographics of patients seen in the complicated *C. difficile* clinic.

Variable	All Patients *n* = 113	FMT *n* = 52	Met Criteria but Not Treated with FMT *n* = 25	Did not meet Criteria for FMT *n* = 36	*p* Value
Age, mean (SD), y	64 (18.5)	67 (16.8)	60 (21.1)	62 (18.8)	0.23
Female sex, N (%)	80 (71)	41 (79)	16 (64)	23 (64)	0.22
Body mass index, mean	26.12	27.04	25.79	25.02	0.29
Body mass index, SD	5.99	6.42	5.82	5.41	
Mean number of *C. difficile* positive stools (SD)	3 (1.50)	3 (1.51)	3 (1.33)	2 (1.80)	0.01
Hospitalized in past year, N (%)	74 (66)	35 (67)	17 (68)	22 (61)	0.80
CDI related hospitalization, N (%)	58 (51)	27 (52)	14 (56)	17 (47)	0.80
Diabetes mellitus, N (%)	23 (20)	10 (19)	6 (24)	7 (19)	0.88
Hypertension, N (%)	65 (58)	30 (58)	13 (52)	22 (61)	0.78
Hyperlipidemia, N (%)	49 (43)	26 (50)	6 (24)	17 (47)	0.08
Gastrointestinal disorders, N (%)	75 (66)	33 (64)	18 (72)	24 (67)	0.76
IBD, N (%)	14 (12)	6 (12)	5 (20)	3 (8)	0.38
Ulcerative colitis, N	6	2	2	2 (6)	
Indeterminate colitis, N	3	2	1	0	
Crohn’s disease, N	4	2	2	0	
Possible IBD, N	1	0	0	1	
Cardiac disorders, N (%)	37 (33)	14 (27)	6 (24)	17 (47)	0.08
Malignancy, N (%)	29 (26)	15 (29)	7 (28)	7 (19)	0.58
Pulmonary disorders, N (%)	28 (25)	7 (14)	7 (28)	14 (39)	0.02
Thyroid disorders, N (%)	25 (22)	12 (23)	3 (52)	10 (28)	0.34
Neurological disorders, N (%)	25 (22)	12 (23)	7 (28)	6 (17)	0.56
Renal disorders, N (%)	21 (19)	11 (21)	4 (16)	6 (17)	0.81
Immunosuppressive medications, N (%)	27 (24)	10 (19)	9 (36)	8 (22)	0.26
Antibiotics (non-CDI), N (%)	79 (70)	38 (73)	17 (68)	24 (67)	0.79
Acid suppressing agents, N (%)	44 (39)	20 (40)	11 (44)	13 (36)	0.82
At skilled nursing facility, N (%)	16 (14)	9 (17)	2 (8)	5 (14)	0.55

**Table 4:** Reasons for not pursuing fecal microbiota transplantation (FMT) in patients with ≥3 *Clostridioides difficile* infections (CDI) recurrences.

Reason for not doing FMT	Numbers
Resolved without FMT	9
Did not follow up in clinic	6
Patient preference	5
Deferred due to risk from procedure due to leukopenia	1
Deferred due to risk from procedure due to congestive heart failure	1
Unable to complete colon prep	1
Moved away	1
Diarrhea attributed to other cause	1

**Table 5:** Gross and microscopic pathology at colonoscopy.

Gross Pathology (*n* =50)	*n* (%)
Normal	11 (22)
Abnormal	39 (78)
Diverticulosis	22 (44)
Gross Inflammation	19 (38)
Erythematous mucosa	10 (20)
Colitis	5 (10)
Ileal ulceration	1 (2)
Pseudomembranes	1 (2)
Mucosal congestion	2 (4)
Angiodysplasia	2 (4)
Neoplasm (new)	5 (10)
Polyp(s)	4 (8)
Mucosal edema	1 (2)
**Microscopic Pathology (*n* = 35)**	**n (%)**
Normal	19 (54)
Abnormal	16 (46)
**IBD**	
Colon: IBD	7
Newly diagnosed IBD	5
Ileum: Ulcers, IBD	1
**Microscopic colitis**	
Collagenous colitis	2
Lymphocytic colitis	2
Focal increase in intraepithelial lymphocytes	1
**Acute Inflammatory Process**	
Mildly active acute colitis	1
Surface mucosal ulceration and epithelial regeneration	1
Mild nonspecific reactive epithelial changes	1
**Neoplastic Process**	
Tubular or tubulovillous adenoma	4
Moderately differentiated adenocarcinoma	1
